# Altered efficacy of AT1R-targeted treatment after spontaneous cancer cell-AT1R upregulation

**DOI:** 10.1186/1471-2407-11-274

**Published:** 2011-06-26

**Authors:** Eleanor I Ager, Shu Wen Wen, Joyna Chan, Way W Chong, Jaclyn H Neo, Christopher Christophi

**Affiliations:** 1 The Department of Surgery, Austin Health, The University of Melbourne, Heidelberg, VIC, Australia

**Keywords:** renin angiotensin system, liver metastases, biomarker, combination therapy

## Abstract

**Background:**

Targeting of the renin angiotensin system (RAS) reduces tumour growth in experimental models of cancer. We aimed to establish if combined targeting of the 'classical' and 'alternative' arms of the RAS could result in synergistic inhibition of colorectal cancer (CRC) liver metastases.

**Methods:**

Immediately following induction of CRC liver metastases through intrasplenic injection of murine CRC cells, treatment with irbesartan (AT1R blocker; 50 mg/kg/day s.c.), captopril (ACE inhibitor; 750 mg/kg/day i.p.), CGP42112A (AT2R agonist; 0.6 μg/kg/hr i.p.), and/or ANG-(1-7) (24 μg/kg/hr i.p.) began and continued for 21 days. Liver to body weight ratio and/or stereology were used as a measure of tumour burden. Immunohistochemistry was used to determine AT1R and VEGF expression as well as proliferation (Ki67), apoptosis (active caspase 3) and angiogenesis (CD34).

**Results:**

Combined RAS therapies failed to improve upon single arm therapies. However, while irbesartan previously inhibited tumour growth in this model, in the current experiments irbesartan failed to affect tumour burden. Subsequent analysis showed a cancer-cell specific upregulation of the angiotensin II type I receptor (AT1R) in irbesartan-insensitive compared to irbesartan-sensitive tumours. The upregulation of AT1R was associated with an increase in proliferation and VEGF expression by cancer cells. While animals bearing irbesartan-sensitive tumours showed a marked decrease in the number of proliferating cells in the liver and VEGF-expressing infiltrating cells in the tumour following AT1R treatment, these were unchanged by treatment in animals bearing irbesartan-insensitive (high AT1R expressing) tumours.

**Conclusions:**

Although the results do not support increased efficacy of combined treatment, they provide intriguing evidence of the importance of RAS expression in determining patient response and tumour growth potential and suggest that components of the RAS could be used as biomarkers to aid in patient selection.

## Background

Metastasis to the liver is the leading cause of death in patients with colorectal cancer (CRC)[1]. We previously showed that targeting of the renin angiotensin system (RAS) with either an angiotensin (ANG) II type I receptor (AT1R) blocker (irbesartan) or an angiotensin converting enzyme (ACE) inhibitor (captopril) could inhibit tumour growth in an orthotopic syngeneic mouse model of CRC liver metastases [2,3]. ACE is responsible for converting inactive ANG I into the key active peptide of the classical RAS, ANG II. The AT1R mediates proliferative, proinflammatory, and angiogenic effects of ANG II [4,5].

The RAS also has an 'alternative' pathway which counteracts many of the actions induced by ANG II-AT1R signalling. The alternative ANG II receptor (the AT2R) generally exerts actions antagonistic to the AT1R including inhibition of proliferation and promotion of apoptosis [6]. ACE2, a homologue of ACE, generates a second RAS peptide, ANG-(1-7), directly from ANG II. ANG-(1-7), through its specific receptor MasR, also appears to counteract many of the actions induced by the classical AT1R/ANGII RAS pathway [7]. Activation of the alternative ANG II receptor, the AT2R, has been shown to inhibit tumour growth (although to lesser extent then either irbesartan or captopril)[5]. ANG-(1-7) can also be infused to reduce tumour growth in several experimental cancer models [8,9]. Two independent Phase I clinical trials are examining ANG-(1-7) [10] and AT1R blockade [11] in the treatment of various solid tumours.

Given the counter-regulatory actions of the classical and alternative RAS pathways we hypothesized that combining inhibition of the classical RAS (AT1R blockade or ACE inhibition) with activation of the alternative RAS (ANG-(1-7) infusion or AT2R activation) would synergistically inhibit tumour growth.

## Methods

### In vivo model and cell lines

The mouse colorectal cancer (MoCR) cell line used for *in vivo *experiments was harvested from a dimethylhydrazine-induced colon carcinoma in a CBA mouse at a stage known to metastasise to the liver [12]. Liver metastases were induced as described previously [3,12]. Briefly, 25000 MoCR cells were injected into the spleen of 6 to 8 week old male CBA mice and, after 3 minutes, the spleen removed to confine metastases to the liver. A minimum of 5 animals per group were used, in treatments inducing fewer tumours sample size was increased to 10. All experiments were approved by the Austin Health Animal Ethics Committee. Liver samples were collected and fixed in fresh 4% PFA.

### Drugs/agents and treatments

*In vivo *treatments included ANG-(1-7) (Auspep, 2588; 24 μg/kg/hr), CGP42112A (AT2R agonist, Sigma-Aldrich, C160; 0.6 μg/kg/hr), and/or telmisartan (AT1R blocker, Sigma-Aldrich, T8949; 12.5 μg/kg/hr) infusion (Alzet^® ^osmotic mini pumps 1004) or s.c. daily injections of irbesartan (AT1R antagonist, Bristol-Myers Squibb) at 50 mg/kg. Captopril was given as daily i.p. injections of 750 mg/kg (Sigma-Aldrich, 21751). Doses were based on previously published studies [3,5,13-15]. The solubilising agent (saline or methyl cellulose) provided a control. Treatments continued from the time of tumour induction to tissue collection at day 21.

### Immunohistochemistry

AT1R (rabbit polyclonal against human, Santa Cruz, sc-1173), proliferation (Ki67; rat monoclonal anti-mouse, Thermoscientific, RM-9106-S1), apoptosis (active caspase 3; rabbit polyclonal anti-human, R&DSystems *AF835*), angiogenesis (CD34, neovascularisation marker; monoclonal rat anti-mouse, Abd Serotec MCA18256), and VEGF (rabbit polyclonal anti-human, CalBiochem, PC315) were assessed in PFA-fixed paraffin embedded tissues. Specificity of AT1R and VEGF antibodies was confirmed by western blot (data not shown). AT1R was used at a concentration of 0.5 μg/ml, Ki67 at 1:100 dilution (dilution provided with manufacturer's datasheet), active caspase-3 at 1.0 μg/ml, CD34 at 0.1 μg/ml, and VEGF at 1.5 μg/ml. Non-immunized rabbit IgG (Santa Cruz, sc-2027) at an equivalent concentration to the primary target antibody was used as a negative control. Endogenous peroxidases were blocked with 3% H_2_O_2 _and non-specific binding inhibited with 10% normal goat serum (Zymed, 01-6201). Slides were incubated with primary antibodies at 37°C for 1 hour and then 4°C overnight. Slides were then incubated with the secondary antibody (Dako Envision^+ ^Goat anti-rabbit HRP secondary *4011 *for AT1R, Ki67, caspase 3, and VEGF and the Rat on Mouse AP-polymer kit (Biocare Medical; RT518H) for CD34 for 1 hour at 37°C before visualisation with DAB or, for CD34, Vulcan fast red (Applied Medical FR805H). Slides were counterstained with Mayer's haematoxylin.

Images of stained tumours were taken using digital light microscope (Nikon Coolscope^®^, Nikon Corporation, Japan) at between 40x and 400x magnification (with a scale bar for size calibration) and were analysed using Image-Pro plus (version 5). Depending on the animal and its tumour load, between 10 and 30 images across 1 to 5 tumours were taken for analysis. AT1R staining was assessed in control tissues, while VEGF, Ki67, caspase3, and CD34 were all examined in control and irbesartan treated tissues. Where possible, the number of positive cells out of the total number of cells was used to measure changes in protein levels/content. A scoring system was used to quantify differences in the strength and abundance of AT1R and VEGF staining in tumours. The intensity of immune-reactive staining was evaluated subjectively (by two independent researchers) using the intensity of immunoreactive colour development as an measure of relative protein content. Strong staining of many/most cells was given a grade of 5 reducing to no staining (0). Only areas of viable tumour were considered in analysis. A subpopulation of intense VEGF expressing infiltrating cells was also counted and is expressed as the number of positive cells per area tumour. Ki67 and active caspase3 were assessed as both the area of proliferating tumour (4x magnification) and the number of proliferating cells per area tumour or liver (20x magnification). CD34 was assessed as the length of positively stained endothelium per tumour area and provide an indication of the angiogenic potential of these tumours.

### Statistical analyses

Quantitative data are presented as mean ± SEM or boxplots showing the minimum value, first quartile, median, third quartile and maximum value. Statistical analyses were conducted using SPSS (Statistical Package for the Social Sciences, version 17, USA) or Microsoft Excel (2003). Normally distributed data were assessed by ANOVA. Mann-Whitney U test were used for non-parametric data sets. A probability (*P*) value of less than 0.05 was considered as statistically significant.

## Results

### Captopril and ANG-(1-7) inhibited tumour burden while AT1R blockade had no significant effect

**Figure 1:**
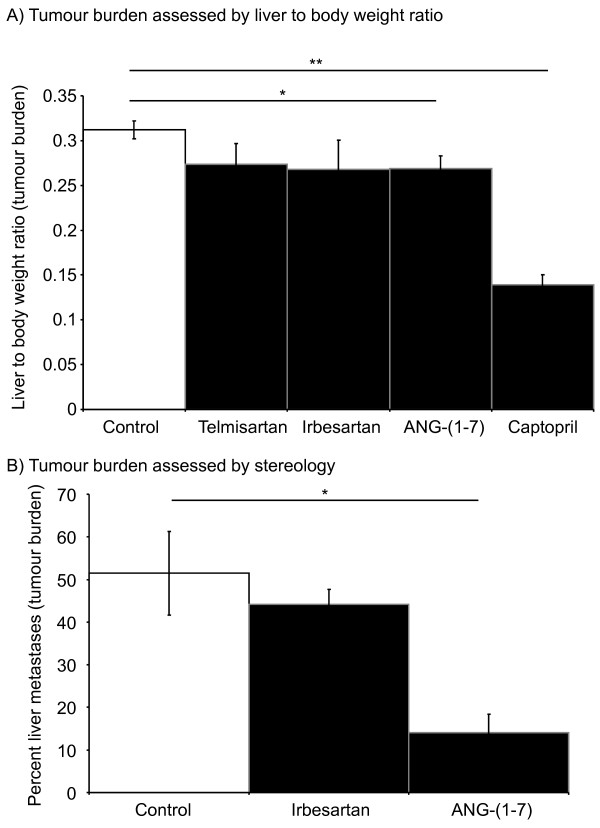
**Liver to body weight ratio 21 days after tumour inoculation with continuous RAS-targeted single treatments (A)**. Percent liver metastases for irbesartan and ANG-(1-7) or control (untreated) animals from the same study as above (B). Telmisartan and irbesartan are AT1R blockers, while captopril is an ANCE inhibitor. n = 5 or 6 for each group. Significant P values between 0.01 and 0.05 are shown with an *, those less than 0.01 are shown with **. Data are presented as mean ± S.E.M.

Mice were induced with CRC liver metastases and treated with irbesartan, ANG-(1-7), or captopril while control received vehicle (PBS or methyl cellulose). Liver-to-body weight ratio was used as a measure of liver tumour burden. No significant difference between the control and the irbesartan treated group was detected (Figure 1A). Given that we had previously found irbesartan inhibited tumour growth in this model [3] we decided to test another AT1R blocker, telmisartan (also presented in Figure 1A); however, again there was no significant difference between control and telmisartan treated groups. We also decided to examine tumour burden by quantitative stereology as this provides a more sensitive measure of tumour load. However, as with the liver to body weight ratio, no significant difference was seen in the response of tumours to either irbesartan (Figure 1B) or telmisartan (data not shown), while we confirmed the ANG-(1-7)-associated reduction in tumour growth (*P *= 0.00263, t-test unequal variance) (Figure 1B).

ACE inhibition with captopril was found to retain its anti-tumour activity (Figure 1A). Captopril-treated animals had significantly lower liver-to-body weight ratios (0.312 ± 0.010) compared to the control group with (0.139 ± 0.011) (*P *= 2.907 x 10^-8^, t-test equal variance). The level of tumour inhibition achieved by captopril treatment in the current experiment is comparable to our previously published studies [2,3].

### Combination therapies targeting the RAS did not improve upon levels of tumour inhibition achieved through single arm therapies

**Figure 2:**
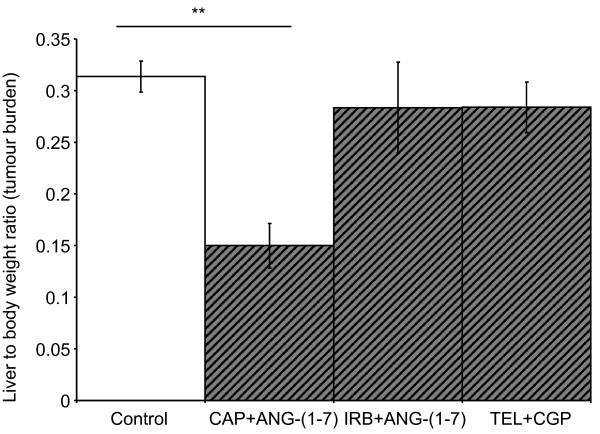
**Liver to body weight ratio 21 days after tumour inoculation with continuous RAS-targeted combined treatments**. CAP, captopril; IRB, irbesartan; TEL, Telmisartan. n = 5 or 6 for each group. Significant P values between 0.01 and 0.05 are shown with an *, those less than 0.01 are shown with **. Data are presented as mean ± S.E.M.

Although irbesartan alone did not significantly inhibit tumour growth, we continued with the initial aim of combining AT1R blockade and/or captopril with ANG-(1-7) infusion. As with the single captopril treatment, combined treatment with captopril and ANG-(1-7) significantly inhibited tumour growth (Figure 2). However, there was no difference between the level of inhibition achieved by captopril alone (liver to body weight ratio of 0.139 ± 0.11) compared to combined captopril plus ANG-(1-7) (liver to body weight ratio of 0.149 ± 0.02). Irbesartan plus ANG-(1-7) failed to decrease tumour burden (Figure 2); this is despite the fact that ANG-(1-7) alone resulted in a reduction in liver to body weight ratio (Figure 1A and 1B). Similarly, AT2R activation with CGP42112A combined with AT1R blockade by telmisartan failed to decrease tumour burden despite previously reported CGP42112A-induced inhibition of CRC liver metastases [5].

### AT1R immunostaining increased in cancer cells from irbesartan-insensitive compared to irbesartan-sensitive animals

While irbesartan was previously found to inhibit tumour growth in this mouse model of CRC liver metastases, in the current experiment, it failed to reduce liver tumour burden despite the same experimental protocols being employed. We hypothesized that this difference was likely due to either to the animals used, which although of the same strain, sex, and age were obtained 2 year apart, or to the cell line, which again may have acquired changes over the intervening years between experiments.

**Figure 3:**
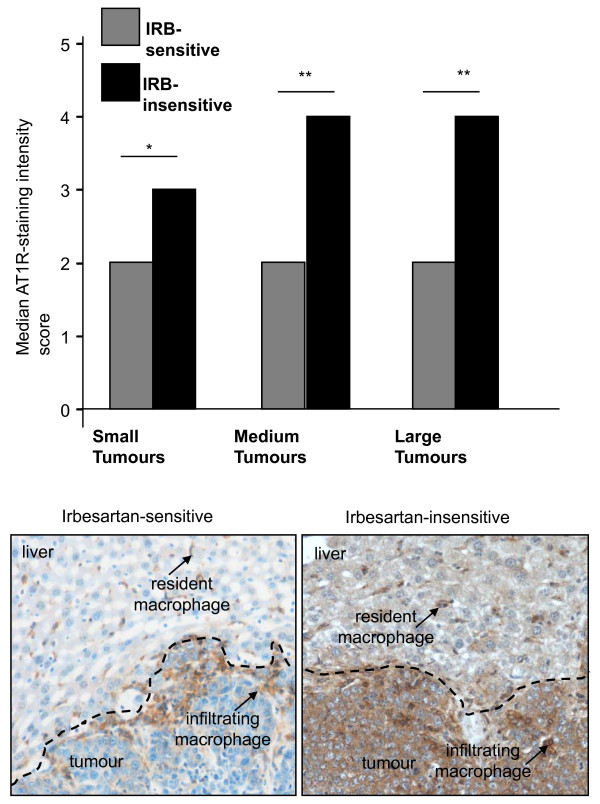
**Intensity of AT1R staining 21 days after tumour inoculation in untreated (control) animals with tumours showing sensitivity to irbesartan or insensitive to irbesartan treatment**. Tumours were grouped into small (≤ 250 μm), medium (250 μm to 1000 μm), or large (≥ 1000 μm) in diameter. Representative images are shown below. n = 5 or 6 for each group except for AT1R^LOW ^tumours which, because of the reduced tumour load, had additional samples (n of 10). The entire tumour was imaged for analysis. Significant P values between 0.01 and 0.05 are shown with an *, those less than 0.01 are shown with **. Data are presented as mean ± S.E.M.

Immunohistochemistry was used to assess the relative level and extent of AT1R expression in CRC-derived tumours growing in the liver from both studies. While no obvious difference was seen in the liver surrounding tumours, tumours from the current study showed a marked upregulation of AT1R expression. AT1R staining in cancer cells was found to be higher in the irbesartan-insensitive animals compared to the irbesartan-sensitive animals (Additional file 1 and Figure 3). Pairwise comparisons between tumours of different sizes all showed higher AT1R expression in irbesartan-insensitive tumours (AT1R^HI^) compared to irbesartan-sensitive tumours (AT1R^LOW^) (small tumours: 3 vs 2, *P *= 0.002; medium tumours score: 4 vs 2, *P *< 0.0001; large tumours: 4 vs 2, *P *< 0.0001 respectively, Mann-Whitney U test).

Within the liver there were AT1R positive cells in both irbesartan-sensitive and irbesartan-insensitive animals. A majority of these cells co-stain with the F4/80 macrophage marker (data not shown). The number and intensity of non-paranchymal, non-cancer cell associated AT1R staining did not appear to differ between the irbesartan-sensitive and the irbesartan-insensitive animals.

### Proliferation of cancer cells was markedly increased in the AT1R^HI ^cancers

**Figure 4:**
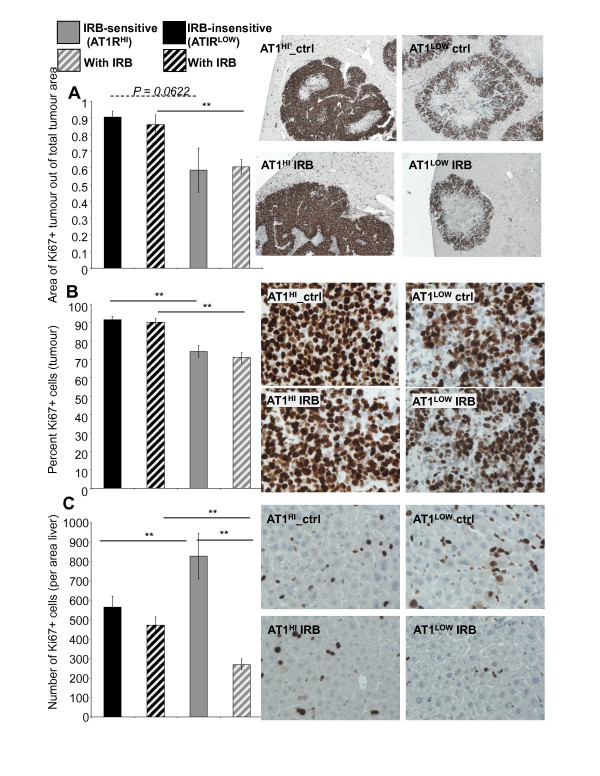
**Area of proliferating tumour 21 days after tumour inoculation in irbesartan sensitive (IRB-sensitive; AT1R^LOW^) and insensitive (IRB-insensitive; AT1R^HI^) tumours either treated with irbesartan (IRB) or untreated.** (A). The percent of Ki67 positive (proliferating) cells per tumour area (measured at 400x magnification) (B). The number of Ki67 positive cells in the liver surrounding tumour (measured at 400x magnification) (C). n = 5 or 6 for each group except for AT1R^LOW ^tumours which, because of the reduced tumour load, had additional samples (n of 10). Between 10 and 30 images were taken from each mouse across 1 to 5 tumours (dependent on tumour load). Representative images for each graph are shown to the right. Significant P values between 0.01 and 0.05 are shown with an * and solid line, those less than 0.01 are shown with **, while those of interest with P values between 0.1 and 0.05 are indicated with a dotted line and no *, but with P value shown. Data are presented as mean ± S.E.M.

AT1R^HI ^tumours were associated with a marked increase (between a 17% to 32% increase) in proliferation compared to AT1R^LOW ^tumours (Figure 4A and 4B). This was significant for all comparisons (*P *≤ 0.0052, t-test) when measured as the number of proliferating cells, but was a trend when measured as the area of proliferation (AT1R^HI ^control compared to AT1R^LOW ^control, Figure 4A). Neither hi- nor low-AT1R-expressing tumours showed a reduction in proliferation with irbesartan treatment. This corresponds with previously unpublished data from our group which showed an increase in apoptosis with irbesartan, but no change in proliferation (Additional file 2).

In contrast to the increase in cancer-cell proliferation by AT1R^HI ^tumours, the liver surrounding these tumours had fewer (*P *≤ 0.0327, t-test) proliferating cells compared to animals bearing AT1R^LOW ^tumours (Figure 4C). Moreover, there was a marked reduction (*P *≤ 0.0002, t-test) in the number of these proliferating cells with irbesartan treatment in the AT1R^LOW^, but not the AT1R^HI ^tumour-bearing animals. It is not clear what type of cell is represented, but both non-paranchymal and paranchymal cells were present in the proliferating population and based on morphology and location relative to the sinusoids, these cells are likely to be hepatic stellate cells or metastasizing tumour cells. Indeed, in our previous study we showed that irbesartan could reduce the number of metastatic lesions [3].

### Apoptosis was increased by irbesartan treatment in both AT1R^HI ^and AT1R^LOW ^tumours

**Figure 5:**
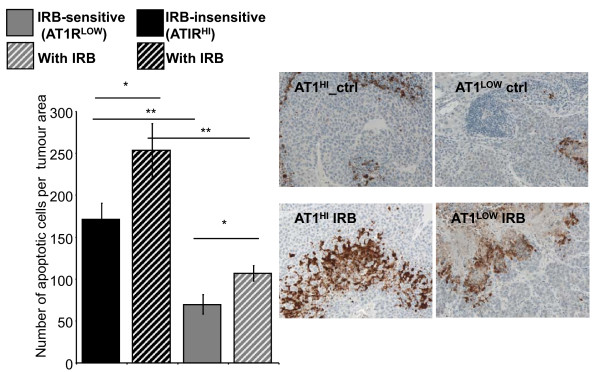
**The number of apoptotic cells per tumour area (measured at 200x magnification)**. n = 5 or 6 for each group except for AT1R^LOW ^tumours which, because of the reduced tumour load, had additional samples (n of 10). Between 10 and 30 images were taken from each mouse across 1 to 5 tumours (dependent on tumour load). Representative images are shown to the right. Significant P values between 0.01 and 0.05 are shown with an *, those less than 0.01 are shown with **. Data are presented as mean ± S.E.M.

Irbesartan treatment significantly increased the number of apoptotic cells in both AT1R^HI ^tumours (*P *= 0.0291, t-test) and AT1R^LOW ^tumours (*P *= 0.0144) (Figure 5). Although this increase was not as great in the AT1R^HI ^tumours compared to that seen in the AT1R^LOW ^tumours. Both treated and non-treated AT1R^HI ^tumours showed higher levels of apoptosis than their corresponding AT1R^LOW ^tumours (*P *≤ 0.000, t-test).

### VEGF expression was higher in AT1R^HI ^compared to AT1R^LOW ^tumours

**Figure 6:**
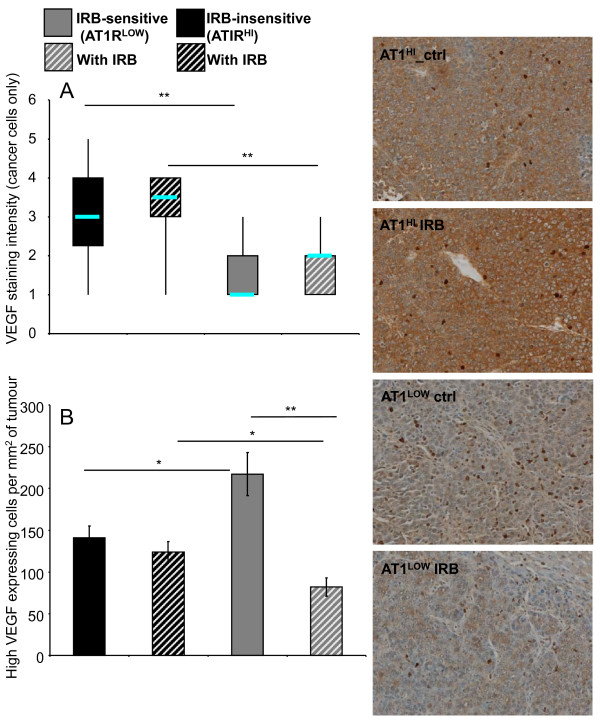
**Intensity of VEGF staining of cancer cells from irbesartan sensitive (Irb-sensitive; AT1R^LOW^) and insensitive (Irb-insensitive; AT1R^HI^) tumours either treated with irbesartan (IRB) or untreated**. (A). Counts of high VEGF-expressing infiltrating cells per tumour area (measured at 200x magnification) (B). n = 5 or 6 for each group except for AT1R^LOW ^tumours which, because of the reduced tumour load, had additional samples (n of 10). Between 10 and 30 images were taken from each mouse across 1 to 5 tumours (dependent on tumour load). Representative images showing both the cancer-cell VEGF staining intensity and the high-staining infiltrating cells are shown to the right. Significant P values between 0.01 and 0.05 are shown with an *, those less than 0.01 are shown with **. Data for A are shown as a box plot to illustrate changes in the median values and range while data for B are presented as mean ± S.E.M.

Similar to cancer-cell proliferation, cancer cell VEGF expression was significantly higher in AT1R^HI ^tumours compared to their corresponding AT1R^LOW ^tumours (*P *≤ 0.000, t-test) (Figure 6A). Interestingly, the median level of VEGF intensity was higher for both irbesartan treated groups (although this failed to reach significance).

While cancer cell-associated VEGF was higher in the AT1R^HI ^tumours (*P *≤ 0.0123, t-test), the number of intense-staining infiltrating cells was highest in the AT1R^LOW ^tumours and this was decreased by irbesartan treatment (*P *= 1.109 x 10^-5^) (Figure 6B).

### CD34+ (angiogenic) vessels were increased in AT1R^HI ^tumours and decreased by irbesartan treatment in both AT1R^HI ^and AT1R^LOW ^tumours

**Figure 7:**
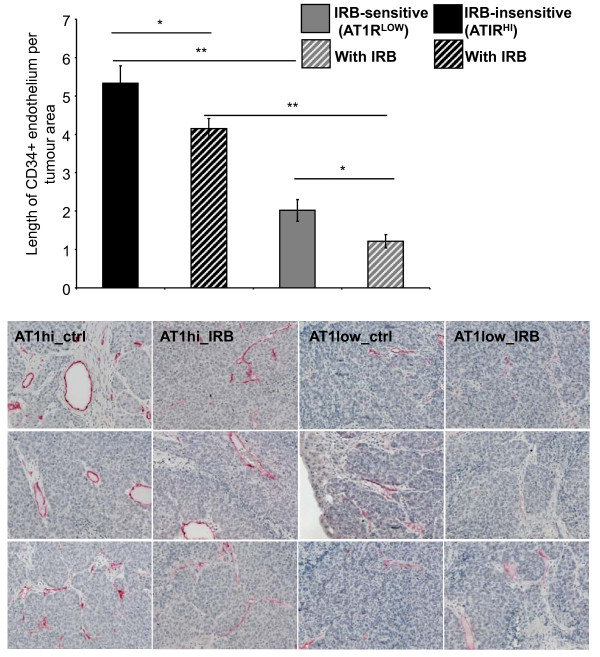
**CD34 staining (measured as the length of CD34-positive endothelium per tumour area) from irbesartan sensitive (IRB-sensitive; AT1R^LOW^) and insensitive (IRB-insensitive; AT1R^HI^) tumours either treated with irbesartan (IRB) or untreated**. n = 5 or 6 for each group except for AT1R^LOW ^tumours which, because of the reduced tumour load, had additional samples (n of 10). Between 10 and 30 images were taken from each mouse across 1 to 5 tumours (dependent on tumour load). Representative images are shown below. Representative images are shown to the right. Significant P values between 0.01 and 0.05 are shown with an *, those less than 0.01 are shown with **. Data are presented as mean ± S.E.M.

Our unpublished data from previous studies (Additional file 3) showed that ANGII infusion increased the length of CD34+ endothelium in tumours while irbesartan decreased CD34+ endothelium compared to tumours from control (untreated) animals. Here we also found that irbesartan decreased the length of CD34+ endothelium in tumours and that this was the case regardless of cancer cell AT1R expression level (Figure 7). Similar to the increase in apoptosis, the decrease in CD34 staining was greatest in the AT1R^LOW ^tumours (*P *= 0.0267 for AT1R^HI ^compared to *P *= 0.0179 for AT1R^LOW ^tumours). However, both treated and non-treated AT1R^HI ^tumours had more angiogenic vessels than AT1R^LOW ^tumours (*P ≤ *0.000, t-test).

## Discussion

Blockade of the classical RAS through AT1R blockade or ACE inhibition reduces tumour growth in several experimental mouse models of cancer [2,3,16,17]. Conversely activation of the alternative RAS, through ANG-(1-7) infusion [8] or AT2R activation [5], can also reduce tumour growth. We sought to determine if greater inhibition of tumour growth could be achieved through dual targeting of the RAS - inhibition of the classical RAS with simultaneous activation of the alternative RAS. While, we were unable to show any benefit for combined RAS treatments, we found that an upregulation of the AT1R by the cancer cell line rendered them insensitive to AT1R blockade. These same cells, however, remained sensitive to ACE inhibition, suggesting significant differences in the molecular mechanisms by which these agents inhibit tumour growth.

The anti-tumour effects of AT1R blockers have been well documented [3,18,19]. However, in our experiment, irbesartan, when used alone or in combination with ANG-(1-7) infusion, failed to decrease tumour growth. We also tested the potent AT1R blocker telmisartan, which has been utilised as an anti-cancer agent in other experimental models where it was shown to be effective [16,20,21], but again we found no benefit of treatment compared to control animals. The lack of an effect of AT1R blockade also contradicts a previous study conducted in our laboratory using the same experimental protocols [3].

To further investigate this finding we performed immunohistochemical analysis on tumours from both studies (for which tissues had been collected in the same experimental manner). AT1R expression by cancer cells from animals insensitive to AT1R blockade showed a marked increase in immunohistochemical staining compared to tissues collected from our previous irbesartan sensitive animals. These results suggest that cancer cells could be rendered insensitive to AT1R blockade through an upregulation of the AT1R. Moreover, despite strong AT1R expression by liver macrophages described here and elsewhere [22] and the importance of macrophages during tumour progression [23], our results suggest that cancer cell AT1R expression can overcome any macrophage-mediated antitumour activity associated with AT1R blockade.

The AT1R is also known to regulate angiogenesis, migration, and apoptosis all of which could contribute to regulating tumour growth [24,25]. Thus, it is likely that the upregulation of AT1R is, at least in part, responsible for the resistance of these cells to AT1R blockade. In support of this we found that high AT1R-expressing tumours had increased cancer cell proliferation, higher levels of cancer cell-associated VEGF and tumour-associated angiogenesis. Interestingly, irbesartan lead to a slight increase in VEGF levels in both tumour types. This is in contrast to some studies of breast cancer [26,27], but supports our previous study which showed that, in these tumours, activation of the AT2R increased VEGF expression [5]. Clere et al. (2010) similarly found that in fibrosarcoma, the AT2R could promote cancer cell VEGF production [28]. AT2R expression has also been documented in blood vessels of human pituitary adenomas [29] and both the AT1R and AT2R stimulate VEGF secretion by rat pituitary tumour cells [30]. Thus, it would appear that in at least some cancer-associated circumstances the AT2R can also be proangiogeneic. However, angiogenesis (as measured by CD34 staining) was decreased by irbesartan and this appeared to be associated with a decrease in the number of infiltrating VEGF-expressing cells.

We also report an increase in apoptosis and a decrease in angiogenic vessel formation associated with irbesartan, and while still present in AT1R^HI ^tumours, these effects were less than that seen for AT1R^LOW ^tumours. Additionally, while it is apparent that cancer-cell AT1R expression has a direct effect on cancer cell proliferation, VEGF-expression, and apoptosis, we also saw marked changes in the surrounding liver and in cells infiltrating the tumours. In particular, irbesartan sensitive tumours (AT1R^LOW^) when untreated (i.e. control animals) were associated with higher numbers of VEGF-infiltrating cells in tumours and higher numbers of proliferating cells in the liver surrounding tumours. Both of these phenomena were reduced by irbesartan treatment. These results would suggest that while tumour resistance was conferred by higher cancer cell-AT1R expression (and that this was associated with certain growth advantages such as increased proliferation and reduced treatment-associated apoptosis), the non-parenchymal cells in the liver may be important in determining the response of low AT1R-expressing tumours to irbesartan.

In contrast to AT1R blockade, ACE inhibition by captopril maintained its effectiveness, consistent to the previous findings [3]. This suggests that ACE inhibitors, other than blocking the production of ANG II which subsequently binds AT1R, also generate anti-tumour effects through non-ANG II mediated pathways. Indeed this has been documented in several studies where matrix metalloproteinases and vascular endothelial growth factor expression have been recognised as non-RAS dependent targets of ACE inhibition [31-33].

## Conclusions

This research initially set out to determine if AT1R blockade or ACE inhibition in combination with either ANG-(1-7) or AT2R activation could lead to synergistic inhibition of CRCLM. While we failed to show any benefit of combined targeting of the RAS, our results were fortuitous in providing insight into both the importance of differences between the mechanisms of action of ACE inhibition and AT1R blockade as well as the potential of RAS expression as a biomarker.

## Abbreviations

ANG II: angiotensin II; ACE: angiotensin converting enzyme; AT1R: angiotensin II type 1 receptor; AT2R: angiotensin II type 2 receptor; CRC: colorectal cancer; VEGF: vascular endothelial growth factor; RAS: renin angiotensin system.

## Competing interests

The authors declare that they have no competing interests.

## Authors' contributions

EA conceived the project aims, performed experimental and statistical analysis and drafted the manuscript. EA, SW, JC, WC, and JN contributed equally to experimental work. CC reviewed the manuscript and aided in the development of the concepts tested. All authors read and approved the final manuscript.

## Pre-publication history

The pre-publication history for this paper can be accessed here:

http://www.biomedcentral.com/1471-2407/11/274/prepub

## Supplementary Material

Additional file 1 **Table indicating the intensity of AT1R staining by different CRC liver metastases**. AT1R staining scores in tumours from animals showing insensitivity to irbesartan treatment and animals in which irbesartan successfully inhibited tumour growth (irbesartan-sensitive). All treatment protocols including agent dose and timing, cancer cell numbers (and type -expecting AT1R expression as described below) and method of induction, as well as tissue collection and processing were identical between experiments. Click here for file

Additional file 2 **Figure showing proliferation and apoptosis of cancer cells from irbesartan-sensitive tumours**. Proliferation of cancer cells from irbesartan-sensitive tumours as measured by PCNA immuno-staining (A). Apoptosis, measured by the percent of active-caspase3-positively stained tumour area, of cancer cells in the same tumour/liver samples as in A (B). Click here for file

Additional file 3 **Angiogenesis (CD34 staining) of irbesartan-sensitive tumours**. Degree of angiogenic vessel formation (as measured by CD34immuno- staining from animals bearing Irbesartan-sensitive tumours Click here for file
