# Supplementary material for: Altered efficacy of AT1R-targeted treatment after spontaneous cancer cell-AT1R upregulation
Source: BMC Cancer. 2011 Jun 26;11:274. doi: 10.1186/1471-2407-11-274 (PMC3141779; doi:10.1186/1471-2407-11-274)
Supplement: Additional file 1 — Table indicating the intensity of AT1R staining by different CRC liver metastases. AT1R staining scores in tumours from animals showing insensitivity to irbesartan treatment and animals in which irbesartan successfully inhibited tumour growth (irbesartan-sensitive). All treatment protocols including agent dose and timing, cancer cell numbers (and type -expecting AT1R expression as described below) and method of induction, as well as tissue collection and processing were identical between experiments. [file 1471-2407-11-274-S1.DOC]

**Additional File 1:** AT1R staining scores in tumours from animals showing insensitivity to irbesartan treatment and animals in which irbesartan successfully inhibited tumour growth (irbesartan-sensitive). All treatment protocols including agent dose and timing, cancer cell numbers (and type –expecting AT1R expression as described below) and method of induction, as well as tissue collection and processing were identical between experiments.

|  | **Small Tumours** | | **Medium Tumours** | | **Large Tumours** | |
| --- | --- | --- | --- | --- | --- | --- |
|  | irbesartan-sensitive | irbesartan-insensitive | irbesartan-sensitive | irbesartan-insensitive | irbesartan-sensitive | irbesartan-insensitive |
|  | 0 | 0 | 1 | 0 | 1 | 0 |
|  | 7 | 1 | 9 | 3 | 5 | 1 |
|  | 5 | 5 | 13 | 3 | 7 | 1 |
|  | 3 | 5 | 6 | 6 | 0 | 6 |
|  | 1 | 6 | 1 | 5 | 0 | 5 |
|  | 0 | 2 | 0 | 6 | 0 | 11 |
|  | 0 | 0 | 0 | 2 | 0 | 0 |
| **Median** | **2** | **3** | **2** | **4** | **2** | **4** |
