# Supplementary figures and images for: Altered efficacy of AT1R-targeted treatment after spontaneous cancer cell-AT1R upregulation
Source: BMC Cancer. 2011 Jun 26;11:274. doi: 10.1186/1471-2407-11-274 (PMC3141779; doi:10.1186/1471-2407-11-274)

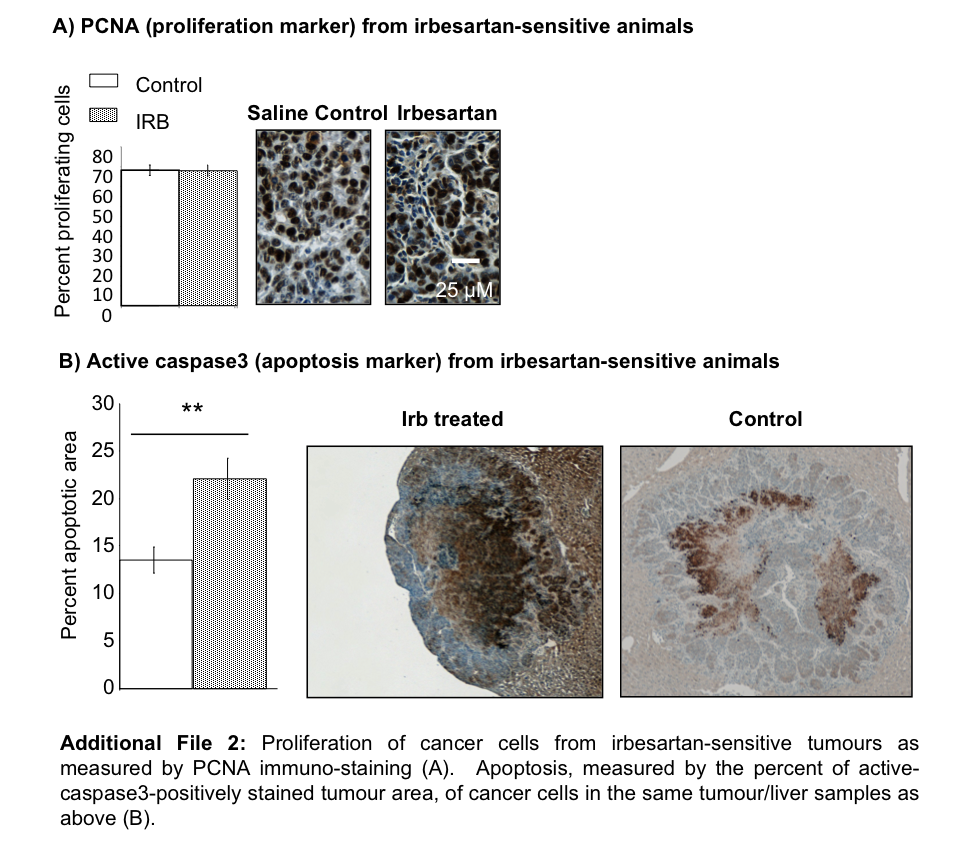

Supplement: Additional file 2 — Figure showing proliferation and apoptosis of cancer cells from irbesartan-sensitive tumours. Proliferation of cancer cells from irbesartan-sensitive tumours as measured by PCNA immuno-staining (A). Apoptosis, measured by the percent of active-caspase3-positively stained tumour area, of cancer cells in the same tumour/liver samples as in A (B). [file 1471-2407-11-274-S2.PNG]

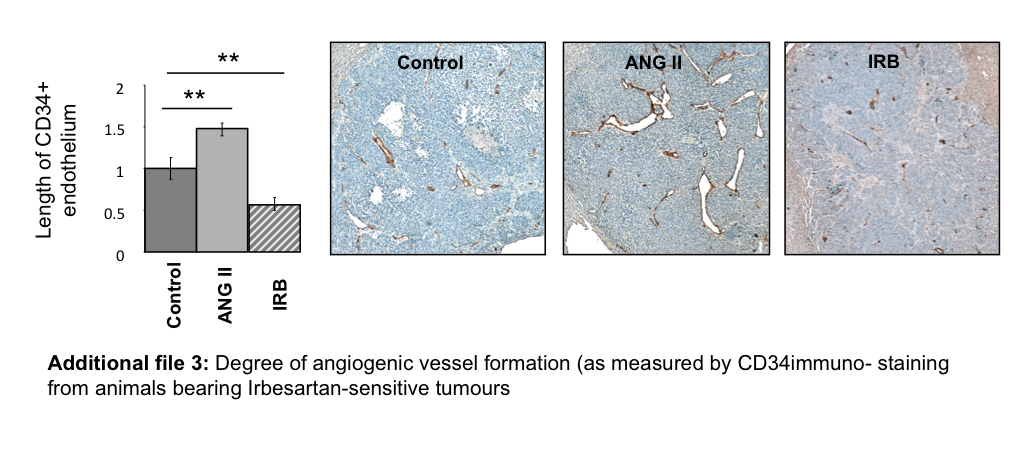

Supplement: Additional file 3 — Angiogenesis (CD34 staining) of irbesartan-sensitive tumours. Degree of angiogenic vessel formation (as measured by CD34immuno- staining from animals bearing Irbesartan-sensitive tumours [file 1471-2407-11-274-S3.PNG]
